# Establishment and application of a quadruple real-time RT-PCR for detecting avian metapneumovirus

**DOI:** 10.1371/journal.pone.0270708

**Published:** 2022-06-28

**Authors:** Suchun Wang, Nan Jiang, Lijian Jiang, Qingye Zhuang, Qiong Chen, Guangyu Hou, Zhiyu Xiao, Ran Zhao, Yang Li, Chenglong Zhao, Fuyou Zhang, Jianmin Yu, Jinping Li, Hualei Liu, Fuliang Sun, Kaicheng Wang

**Affiliations:** 1 China Animal Health and Epidemiology Center, Qingdao, Shandong, China; 2 Agricultural College, Yanbian University, Yanji, Jilin, China; 3 Shandong Vocational Animal Science and Veterinary College, Weifang, Shandong, China; 4 Xiamen Agriculture Product Quality and Safety Test Centre, Xiamen, Fujian, China; University of Illinois College of Medicine, UNITED STATES

## Abstract

In order to develop an appropriate method for high-throughput detection of avian metapneumovirus, a quadruple real-time reverse-transcription polymerase chain reaction assay was established with four pairs of specific primers and four specific probes based on the G or M gene of aMPV-A, aMPV-B, aMPV-C and aMPV-D. Its specificity and sensitivity were evaluated, and clinical samples were tested by the method. The results showed that all the four subgroups of avian metapneumovirus can be detected in the quadruple real-time RT-PCR assay simultaneously, with a detection limit of 100–1000 cRNA copies/reaction. The other common poultry viruses were negative. In the avian clinical sample detection, 39 out of 1920 clinical samples collected from 8 provinces were positive. Compared with published RT-PCR assays, the κ value of the quadruple real-time RT-PCR assay in 1920 avian clinical samples was 1.000 (*P* < 0.001). The established method could be used for the rapid detection of the four subgroups of avian metapneumovirus with high specificity and high sensitivity.

## Introduction

Avian metapneumovirus (aMPV) has been detected for a long time [1]. aMPV can cause both respiratory and reproductive disorders in a variety of poultry, including turkeys, chickens, guinea fowls and pheasants [2]. Four subgroups exist based on the antigenic and molecular variations, termed aMPV-A, aMPV-B, aMPV-C and aMPV-D [3–5]. aMPV was first discovered in South Africa in 1970, spread throughout Europe in the 1980s and was found in the United States in 1996. In recent years, the detection of aMPV has been reported in many countries or regions, especially in Europe [6–12].

aMPV was found in China in 1999, but the subgroup was not identified. Subsequently, many researchers carried out a series of surveys on chicken infection with aMPV. In 2007 and 2008, serological tests were carried out on breeder flocks in some areas of Shandong province, and the results showed that the seropositive rate of several flocks reached 100% [13]. In 2015, 296 serum samples were collected from 9 chicken farms in China, and the observed aMPV seropositivity rate was 74.3% [14]. Multiple survey data indicated that aMPV has become prevalent among chickens in China. However, only aMPV-C was isolated from broilers in southeastern China [10] and Muscovy ducks [15]. aMPV-B was isolated from chickens in Liaoning province [16], whereas aMPV-A was detected using reverse transcription-polymerase chain reaction (RT-PCR) [17]. aMPV-D was not detected in China.

The detection methods of aMPV include virus isolation, enzyme-linked immunosorbent assay (ELISA) and RT-PCR [18–20]. Virus isolation is not generally used as a routine detection for aMPV because of its difficult. aMPV could not be subtyped using Some established ELISA methods. Moreover, these methods are time-consuming and insensitive, and are not suitable for rapid detection of aMPV. Some real-time RT-PCR methods have also been established which could not detected all subtypes of aMPV [21]. In order to improve the detection methods of aMPV and clarify the impact of aMPV on poultry in China, a quadruple real-time RT-PCR was established in this study, which can detect all four subgroups of aMPV simultaneously. The method was evaluated using 1920 avian clinical samples from 8 provinces of China.

## Materials and methods

### Ethics approval and consent to participate

This study was conducted according to the animal welfare guidelines of the World Organization for Animal Health and approved by the Animal Welfare Committee of the China Animal Health and Epidemiology Center. The approval number was DWFL202104.

### Sample collection and RNA extraction

The aMPV-B positive sample was donated by Zhejiang Agriculture & Forestry University, and the aMPV-C positive sample was donated by the Shanghai Animal Disease Prevention and Control Center. Avian influenza virus (AIV), Newcastle disease virus (NDV), infectious bronchitis virus (IBV) and infectious laryngotracheitis (ILTV) were all conserved in the China Animal Health and Epidemiology Center. The 1920 cloacal/throat double swabs were obtained from 25 live poultry markets or farms in 8 provinces of China according to a random sampling method. Live poultry markets or farms of 8 provinces in China were selected at random in 2019. The 1920 samples were collected from five animal species, including white pigeon, goose, chicken, duck, and partridge. The samples were stored in 0.5 mL phosphate-buffered saline (PBS, pH 7.2) containing 10% glycerol at 4°C to 8°C. Viral RNA was extracted within 3 days after collection. The remaining samples were stored at −80°C. The samples were centrifuged at 12,000 *g* at 4°C for 5 min. Viral RNA was extracted according to the manufacturer’s instructions of the cador Pathogen 96 QIAcube HT kit (Qiagen, Hilden, Germany) and stored at −80°C.

### Generation of plasmid standard

Four standard plasmids were developed based on the gene sequence of the aMPV reference strain (GenBank accession numbers AM778585.1, JN651918.1, HG934339.1 and FJ195333.1). The fragments of the whole glycoprotein (G) genes of aMPV-A, aMPV-B and aMPV-D are 1193, 1263 and 1187, respectively. The G genes were cloned into the *pEASY*–T5 Zero Cloning vector. An 870-bp fragment of the whole matrix protein (M) gene of aMPV-C was cloned into the *pEASY*–T5 Zero Cloning vector. Recombinant plasmid DNA was extracted using a SanPrep Column Plasmid Mini-Prep Kit (Sangon, Shanghai, China).

### Synthesize RNA in vitro

The four standard plasmids were digested by enzyme (*Spe*Ⅰand *Not*Ⅰ) and purified with the DNA Extraction Kit (Solarbio, Beijing, China) respectively. The purified DNA was used as template for in vitro transcription reactions with RiboMAX^TM^ Large Scale RNA Production System–SP6 and T7 kit (Promega, USA). The mixture of transcription reaction in vitro include 20 μL of 5× T7 Transcription Buffer, 7.5 μL of rNTPs (25mM ATP, CTP, GTP, UTP), 10 μL T7 Enzyme mix, 30 μL Linear DNA template and 32.5μL Nuclease Free H_2_O. The transcription program was 37°C for 5 h. In order to remove the DNA template, 10 μL RQ1 RNase-Free DNase was added to the reaction system and incubated at 37°C for 15 min. Then, the miscellaneous proteins and various ions in the system were removed with the Rneasy MiNi Kit (Qiagen, Hilden, Germany), and the cRNA was extracted and purified. The cRNA was quantified using a Thermo Scientific Multiskan GO Microplate Photometer (Thermo Fisher Scientific, USA). The RNA copy number was calculated according to the following formula: RNA copy number per μL = (6.02 × 10^23^ × plasmid concentration (ng/μL) × 10^−9^)/(RNA length × 340).

### aMPV real-time RT-PCR primers and probe design

aMPV-A, aMPV-B and aMPV-D specific primers and probes were designed based on the G gene of aMPV. The M gene was selected as reference sequence for aMPV-C for primer and probe design. A total of 36 available G gene sequences of aMPV-A, 36 available G gene sequences of aMPV-B, 40 available M gene sequences of aMPV-C and 3 available G gene sequences of aMPV-D from NCBI GenBank database were aligned to check the specific and conserved regions with Molecular Evolutionary Genetics Analysis (MEGA) software 7.0 respectivly. Multiple primers and probes were designed for each subgroup. The design and evaluation of these primers and probes were performed using Oligo7 software [22]. The primers and probes specific to each subgroup of aMPV were grouped and tested to select the optimal primers and probes. When screening primers and probes, the risk of cross-reactivity issues for different subgroups should be avoided. The TaqMan hydrolysis probes were labeled with fluorescence reporter dye (ROX, FAM, HEX, and CY5) at the 5′-end and quenched with BHQ1 or BHQ2 at the 3′-end. Details of the final primers and exo-probes are shown in Table 1. All primers and exo-probes were synthesized by Sangon.

**Table 1:** Primers and probe sequences used for real-time RT-PCR and RT-PCR.

Primer	Sequence(5^,^-3^,^)	Size(bp)	Gene	subgroup	Source
qRT-PCR					
aMPV-A-F	ACTATATATGGTTCAGGGCAC	21	G	A	This study
aMPV-A-R	TAGTTTCTGCACTCCTCTAACACT	24	G		This study
aMPV-A-P	ROX-CACAGTCACTATTGCACTCACT-BHQ2	22	G		This study
aMPV-B-F	AATAGTCCTCAAGCAAGTCCTCA	23	G	B	This study
aMPV-B-R	GTAATTTGACCTGTTCYACACT	22	G		This study
aMPV-B-P	FAM-CCTTAGGCTTGACGCTCACTA-BHQ1	21	G		This study
aMPV-C-F	TACCAGCCTATATAAAGTCTGT	22	M	C	This study
aMPV-C-R	AGTTATAGCTTGATCTGCC	19	M		This study
aMPV-C-P	HEX-CCCACTAATTGCAGCTTCAACA-BHQ1	22	M		This study
aMPV-D-F	CTCTTCCAAGGGGCTGTGATAA	22	G	D	This study
aMPV-D-R	GCAGCGCTGGTTATAGCCAA	20	G		This study
aMPV-D-P	CY5-ATTATGTGTGTGGATGCCTA- BHQ2	20	G		This study
RT-PCR					
Ga	CCGGGACAAGTATCTCTATGG	21	G	A	[18]
G2	CCACACTTGAAAGATCTACCC	21	G		[18]
Ga	CCGGGACAAGTATCTCTATGG	21	G	B	[18]
G12	CAGTCGCCTGTAATCTTCTAGGG	23	G		[18]
C1	GCGCAACTACCTGCAAGGTTAACAGTAT	28	M	C	[18]
C2	CTTTCCAACTGCCTTGGCTGAATCG	25	M		[18]
G150	CCGATGCCCAGTTAATAA	18	G	D	[18]
G1005	CCCCTTACAAACACTGTTC	19	G		[18]

### Condition optimization

The reaction condition of the quadruple real-time RT-PCR was optimized, including the concentration of primers and probes, temperature and time of annealing/extension. All primers and probes were dissolved to 10 pmol/μL. The optimum reaction concentration of primers and probes were selected by different volumes (list in S2 Table). Five annealing/extension temperature (58°C, 59°C, 60°C, 61°C, 62°C) and three annealing/extension time (20s, 30s, 40s) were tested to screen the best annealing/extension conditions.

### Analytical sensitivity of the quadruple real-time RT-PCR

The analytical sensitivity of each component in the quadruple real-time RT-PCR was evaluated using a tenfold serial dilution of standard recombinant plasmids for the four subgroups (10^6^–10^1^ cRNA copies per reaction). Evaluation of the analytical sensitivity of the quadruple real-time RT-PCR method was performed by detecting each dilution in eight replicates.

### Analytical specificity of the quadruple real-time RT-PCR

The specificity of the quadruple real-time RT-PCR for aMPV was evaluated using one aMPV-B, one aMPV-C, 12 AIVs, one NDV, 1 ILTV and two IBVs. The details of all the virus are listed in Table 2.

**Table 2:** Samples tested in the present study.

Sample	Virus	subtype
aMPV-B	aMPV	B
aMPV-C	aMPV	C
Q232	AIV	H1N2
X2057	AIV	H3N8
S82	AIV	H11N2
T55	AIV	H10N2
GX2032	AIV	H6
H9	AIV	H9N2
P174	AIV	H4N2
K144	AIV	H5N1
G2324	AIV	H5N6
QD-1	AIV	H5N2
H7N3	AIV	H7N3
1605	AIV	H7N9
ND	NDV	/
8	ILTV	/
M41	IBV	/
H52	IBV	/

### Evaluation of the quadruple real-time RT-PCR assay using avian clinical sample

In total, 1920 avian double swabs were detected and compared with the published RT-PCR assay for the four subgroups of aMPVs to evaluate the performance of the quadruple real-time RT-PCR assay. The primers for the published RT-PCR assay [18] are listed in Table 1. According to the published methods, RT-PCR for the four subgroups of aMPVs was performed using the PrimeScript ™ One-step RT-PCR Kit Ver. 2 (Takara, Dalian, China; RR055A), and the reaction system included 12.5 μL of 2× One-step buffer, 1 μL of each primer (10 pM), 1 μL of PrimeScript 1 Step Enzyme Mix, 3 μL of nucleic acids, and 6.5 μL of RNase-free H_2_O. The reaction conditions were as follows: 30 min at 50°C, 4 min at 95°C and 40 cycles of 98°C for 30 s, 58°C for 30 s and 72°C for 30 s, followed by a final extension step for 7 min at 72°C. Then the amplified DNA fragments were sequenced using synthetic oligonucleotides (Sangon Biotech, Shanghai, China). To verify the positive results by conventional PCR, the sequences of the PCR products were compared with the GenBank nucleotide database by online BLAST search at the NCBI website.

## Result

### Quadruple real-time RT-PCR

The quadruple real-time RT-PCR reaction system was performed according to the optimal conditions and programs. The results of the optimal conditions and programs were showed in S3–S6 Tables. The reaction mixture contained the following: 25.0 μL of 2× One Step RT-PCR Buffer Ⅲ, 1.0 μL of 5 U/μL TaKaRa Ex Taq HS, 1.0 μL of PrimeScript RT Enzyme Mix Ⅱ, 1.0 μL of the primers and probes for each subgroup of aMPV (10 pmol/μL), 4.0 μL of RNA template and RNase Free dH_2_O to a final volume of 50.0 μL. The detection program was as follows: 42°C for 5 min and 95°C for 30 s, followed by 40 cycles of 95°C for 5 s and 60°C for 30 s. Cycle threshold (Ct) values were used, as Ct indicates the PCR cycle number at which the amount of amplified target reaches a fixed threshold.

### Sensitivity of the quadruple real-time RT-PCR assay

**Fig 1:**
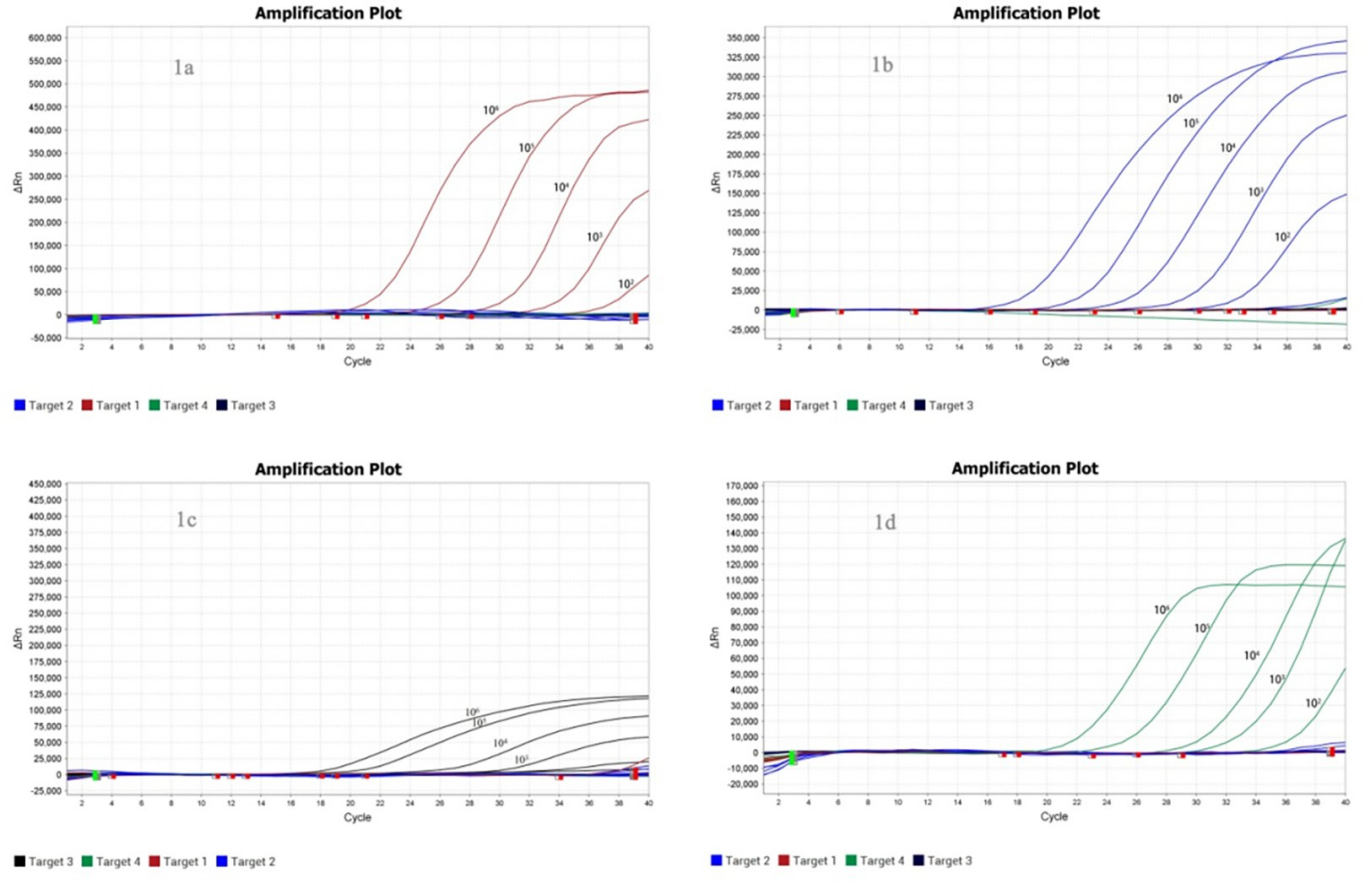
Sensitivity test results of the quadruple real-time RT-PCR diagnostic method for aMPV. (a) Sensitivity test results of the quadruple real-time RT-PCR diagnostic method for aMPV-A. (b) Sensitivity test results of the quadruple real-time RT-PCR diagnostic method for aMPV-B. (c) Sensitivity test results of the quadruple real-time RT-PCR diagnostic method for aMPV-C. (d) Sensitivity test results of the quadruple real-time RT-PCR diagnostic method for aMPV-D.

The sequences of the quadruple real-time RT-PCR primers and TaqMan probes are listed in Table 1. The aMPV-A, aMPV-B and aMPV-D molecular plasmid standards were positive in the assays from 10^3^ RNA copies per reaction. Six, four and four of eight replicates with 10^2^ RNA copies per reaction tested positive in the quadruple real-time RT-PCR assay. For aMPV-C, reactions were positive from 10^4^ copies per reaction. Five of eight replicates with 10^3^ RNA copies per reaction tested positive in the quadruple real-time RT-PCR assay. The detection limits of the quadruple real-time RT-PCR assay at 95% were 10^2^ RNA copies per reaction for aMPV-A, aMPV-B and aMPV-D and 10^3^ RNA copies per reaction for aMPV-C (probit analysis, *P* < 0.05). The detection results of the aMPV-A, aMPV-B, aMPV-C and aMPV-D standards are shown in Table 3 and Fig 1, respectively.

**Table 3:** Assay data used for probit analysis to calculate the detection limits of aMPV-A, aMPV-B, aMPV-C and aMPV-D.

Copies per reaction	No. of positive samples/ No. of samples tested by the quadruple real-time RT-PCR			
	aMPV-A	aMPV-B	aMPV-C	aMPV-D
10^6^	8/8	8/8	8/8	8/8
10^5^	8/8	8/8	8/8	8/8
10^4^	8/8	8/8	8/8	8/8
10^3^	8/8	8/8	8/8	8/8
10^2^	3/8	4/8	0/8	2/8
10^1^	0/8	0/8	0/8	0/8

### Specificity of the quadruple real-time RT-PCR

**Fig 2:**
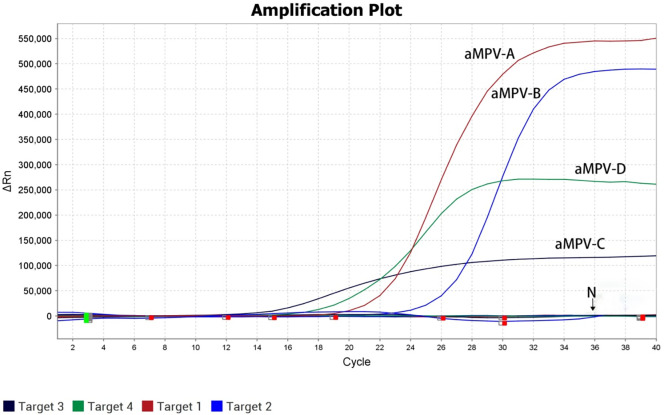
Specificity test results of the quadruple real-time RT-PCR diagnostic method for aMPV.

The quadruple real-time RT-PCR assay was positive for the plasmid of four aMPV subgroups and negative for the samples, including AIVs, NDVs, IBVs and negative controls. aMPV-B and aMPV-C positive samples also tested positive in the quadruple real-time RT-PCR assay, but they tested negative to the probes for aMPV-A and aMPV-D. Thus, the quadruple real-time RT-PCR assay demonstrated high specificity for the detection of all subgroups of aMPV (Fig 2).

### Evaluation of the real-time RT-PCR assay using avian clinical samples

The real-time RT-PCR assay detected 39 positive samples out of 1920, in agreement with the results of the RT-PCR assay. The sensitivity of the RT-PCR was tested by the four standard plasmids. The κ value for RT-PCR and real-time RT-PCR was 1.000 (*P* < 0.001) (Table 4).

**Table 4:** Detection results of aMPV in avian clinical samples.

		real-time RT-PCR		Total	Kappa (κ)	P-value of kappa	Sensitivity%	Specificity%
		Positive	Negative					
RT-PCR	Positive	39	0	39	1.000	<0.001	100 (88.83–100)	100 (99.75–100)
	Negative	0	1881	1881				
Total		39	1881	1920				

### Epidemiological analysis of aMPV

Out of 1920 avian clinical samples, 39 (2.03%) were positive. The geography and host distribution of the 39 positive samples were analyzed. Whenever one or more sample from a live poultry market was tested positive, it would be recorded as a positive site. 1920 avian clinical samples were collected from 25 live poultry markets. The 39 aMPV positive samples were detected in 10 live poultry markets with a group positive rate of 40% (10/25). The distribution of the 39 positive samples in each live poultry market is shown in Table 5. The 22 positive samples which were collected from chickens are aMPV-B. Only one positive sample which was collected from goose is aMPV-C. 4 aMPV-B positive samples and 6 aMPV-C positive samples were collected from ducks. Co-infection of aMPV-B and aMPV-C have not been deteted. The number of samples collected for each animal and the number of samples which tested positive for aMPV are shown in Table 6.

**Table 5:** The distribution of 39 positive samples in each live poultry market.

	Group	No. Of Sample	aMPV-B (Individual Positive Rate)	aMPV-C (Individual Positive Rate)
Anhui	1	30		
	2	60		
	3	150	1 (0.67%)	
Guangdong	1	150		
	2	30		
	3	30		1 (3.33%)
	4	30		
Guangxi	1	30		1 (3.33%)
	2	150		
	3	30		
	4	30		2 (6.67%)
Hebei	1	240		
Hubei	1	120	1 (0.83%)	
	2	120		
Jiangsu	1	45		
	2	75	3 (4.00%)	
	3	120		
Jiangxi	1	150	9 (6.0%)	3 (6.0%)
	2	10		
	3	30		
	4	30		
	5	20	10 (50.00%)	
Shanghai	1	30	1 (3.33%)	
	2	30		
	3	180	7 (3.89%)	
Total	25	1920	32	7

**Table 6:** The host distribution of 39 positive samples of aMPV.

Animal Species	No. of Sample	aMPV-B (Individual Positive Rate)	aMPV-C (Individual Positive Rate)
goose	40		1 (2.5%)
pigeon	235		
chicken	1221	28 (2.29%)	
duck	419	4 (0.95%)	6 (1.43%)
partridge	5		
Total	1920	32	7

## Discussion

aMPV-A, aMPV-B and aMPV-C have been detected in China with various methods [16, 17, 23]. aMPV-D has never been found in China. **aMPV vaccine has not been used in China.** In order to prevent spreading of the disease and facilitate treatment, early detection of viral infection, especially the establishment of a rapid and accurate detection method, has received much attention.

Most real-time PCR detection technologies employ the TaqMan fluorescent probe method. Compared with the traditional SYBR Green method, this method is highly specific. In this study, an aMPV quadruple real-time RT-PCR detection method was established using TaqMan fluorescent probes, which can quickly and accurately detect aMPVs and can be used for large-scale detection of aMPV infection in poultry flocks.

Subtyping of aMPV was based on G gene. The G gene sequences of all aMPV subgroups were downloaded from NCBI. Through homology comparison, it was found that the G gene sequence of aMPV-C was poorly conserved, so the more conserved M protein gene sequence was used as a template to design primers and probes for aMPV-C detection. The G genes of aMPV-A, aMPV-B and aMPV-C are more conserved, and can be used to design detection primers and probes. Therefore, the quadruple real-time RT-PCR detection method was established based on the G genes of aMPV-A, aMPV-B and aMPV-D and the M genes of aMPV-C. The aMPV detection method was developed based on quadruple real-time RT-PCR, while genotyping was performed simultaneously. Compared with RT-PCR and virus isolation, it can reduce the probability of contamination, it can yield experimental results in a short time and it has higher specificity and sensitivity. The nucleic acid samples of aMPV-B and aMPV-C were positive in the quadruple real-time RT-PCR assay. All aMPV positive samples and four standard plasmids used for the evaluation of the quadruple real-time RT-PCR assay were positive. Due to the limitation of the source of aMPV-A and aMPV-D in China, it is currently impossible to test the ability of this method to detect these two subgroups with positive virus samples. However, according to the primer/exo-probe design method and the plasmid standard detection results, the quadruple real-time RT-PCR assay is considered to be suitable for the detection of aMPV-A and aMPV-D.

In order to evaluate the quadruple real-time RT-PCR assay and investigate the prevalence of aMPV in China, 1920 double swabs of live poultry trading markets in eight provinces of Shanghai, Anhui, Jiangxi, Jiangsu, Hubei, Hebei, Guangxi and Guangdong were collected. A total of 39 samples were found to be positive, including 32 aMPV-B and seven aMPV-C samples. The clinical samples tested positive by the quadruple real-time RT-PCR were also confirmed to be positive by PCR detection and sequencing. The test results showed that 28 aMPV-B positive samples were from chicken and four aMPV-B positive samples were from duck. Moreover, six duck samples and one goose sample tested positive for aMPV-C. It can be seen that aMPV-B is common in chickens in China, and aMPV-C is common in ducks. aMPV-C is often reported in geese abroad [24–26]. In our study, aMPV-C in geese was discovered for the first time in China. According to the distribution of the aMPV positive rate, Jiangxi province occupies 9.2%, Shanghai 3.3%, Jiangsu and Guangxi 1.3% and Anhui, Hubei and Guangdong provinces each 0.4%, and aMPV was not detected in Hebei province. This clinical sample test proves that most aMPV infection in chickens in China are subgroup B, and a small number of infected ducks and goose have subgroup C. Effective measures should be taken to prevent spreading of the disease and avoid economic losses.

## Conclusion

The quadruple real-time RT-PCR was established in this study can detect all four subgroups of aMPV simultaneously, is not time-consuming and has good specificity and high sensitivity. This assay provides a powerful tool for the detection of aMPVs, which is important for the control of aMPV.

## Supporting information

S1 Table The sensitivity test results of the RT-PCR method for aMPV. The plasmid of aMPV-A, aMPV-B, aMPV-C and aMPV-D were diluted series for the sensitivity tests. The number of copies per reaction was counted which could be tested positive. The minimum number of copies per reaction was considered to the sensitivity for the four subgroup of aMPV.
(DOCX)

S2 Table The 81 combinations of primers and probes with different volume in the reaction mixture. (DOCX)

S3 Table The test results of different primers and probes volume in the reaction mixture. The quadruple real-time RT-PCR reaction system was performed according to the BioScript (Dalian) Co., Ltd. PrimeScriptTM One Step RT-PCR Kit instructions. The standard recombinant plasmids for the four subgroups (10^4^ cRNA copies per reaction) were used as templates. The mean values of CT were calculated to analyze and screen the optimal volume of primers and probes.
(DOCX)

S4 Table The mean values of CT. The results showed that the mean values of CT were decreasing with the increasing of primer and probes volumes. However, when the volume of primers and probes increased from 1.0μL to 1.4μL, the CT values dose not decreased significantly. Therefore, considering the cost and results of the experiment, the volume of primers and probes for the four subgroups were set as 1.0μL in the 50μL reaction mixture. The reaction mixture contained the following: 25.0 μL of 2× One Step RT-PCR Buffer Ⅲ, 1.0 μL of 5 U/μL TaKaRa Ex Taq HS, 1.0 μL of PrimeScript RT Enzyme Mix Ⅱ, 1.0 μL of the primers and probes for each subgroup of aMPV (10 pmol/μL), 4.0 μL of RNA template and RNase Free dH_2_O to a final volume of 50.0 μL.
(DOCX)

S5 Table The test results of optimum annealing/extension temperature for the quadruple real-time RT-PCR. Five annealing/extension temperature (58°C, 59°C, 60°C, 61°C, 62°C) were tested to screen the optimal annealing/extension conditions. The results showed that CT values were smaller and the sensitivity was higher than other temperatures when the annealing/extension temperature was 59°C or 60°C. In the appropriate temperature range, the specificity of primers and probes will increase with the raising of annealing/extension temperature. Therefore, the annealing/extension temperature of the quadruple real-time RT-PCR was set as 60°C.
(DOCX)

S6 Table The results of optimum annealing/extension time for the quadruple real-time RT-PCR. Three annealing/extension time (20s, 30s, 40s) were tested to screen the optimal annealing/extension conditions. The results showed that there was no significant difference in the CT values when the annealing/extension time was 20s, 30s and 40s respectively. The annealing/extension time was set as 30s to prevent the influence of stability of the experiment by too short annealing/extension time. The quadruple real-time RT-PCR cycler conditions were as follows: 30 min at 50°C, 4 min at 95°C and 40 cycles of 98°C for 30 s, 60°C for 30 s and 72°C for 30 s, followed by a final extension step for 7 min at 72°C.
(DOCX)
