# Correction: Establishment and application of a quadruple real-time RT-PCR for detecting avian metapneumovirus

**DOI:** 10.1371/journal.pone.0294298

**Published:** 2023-11-07

**Authors:** Suchun Wang, Nan Jiang, Lijian Jiang, Qingye Zhuang, Qiong Chen, Guangyu Hou, Zhiyu Xiao, Ran Zhao, Yang Li, Chenglong Zhao, Fuyou Zhang, Jianmin Yu, Jinping Li, Hualei Liu, Fuliang Sun, Kaicheng Wang

An additional affiliation is missing for the last Author. Kaicheng Wang is also affiliated with: Key Laboratory of Animal Biosafety Risk Prevention and Control (South), Ministry of Agriculture and Rural Affairs, Qingdao, Shandong, 266032, P.R. China.
