# Supplementary material for: Establishment and application of a quadruple real-time RT-PCR for detecting avian metapneumovirus
Source: PLoS One. 2022 Jun 28;17(6):e0270708. doi: 10.1371/journal.pone.0270708 (PMC9239461; doi:10.1371/journal.pone.0270708)
Supplement: S1 Table — The plasmid of aMPV-A, aMPV-B, aMPV-C and aMPV-D were diluted series for the sensitivity tests. The number of copies per reaction was counted which could be tested positive. The minimum number of copies per reaction was considered to the sensitivity for the four subgroup of aMPV. (DOCX) [file pone.0270708.s001.docx]

**S1 Table The sensitivity test results of the RT-PCR method for aMPV**

| Subgroup of aMPV | primer | sensitivity (Copies per reaction) |
| --- | --- | --- |
| aMPV-A | Ga | 10^3^ |
|  | G2 |  |
| aMPV-B | Ga | 10^4^ |
|  | G12 |  |
| aMPV-C | C1 | 10^4^ |
|  | C2 |  |
| aMPV-D | G150 | 10^4^ |
|  | G1005 |  |

The plasmid of aMPV-A, aMPV-B, aMPV-C and aMPV-D were diluted series for the sensitivity tests. The number of copies per reaction was counted which could be tested positive. The minimum number of copies per reaction was considered to the sensitivity for the four subgroup of aMPV.
