# Supplementary material for: Establishment and application of a quadruple real-time RT-PCR for detecting avian metapneumovirus
Source: PLoS One. 2022 Jun 28;17(6):e0270708. doi: 10.1371/journal.pone.0270708 (PMC9239461; doi:10.1371/journal.pone.0270708)
Supplement: S2 Table — (DOCX) [file pone.0270708.s002.docx]

**S2 Table The 81 combinations of primers and probes with different volume in the reaction mixture**

| number | Volume of each component (µL) | | | | | | | | | | | | | | | | | |
| --- | --- | --- | --- | --- | --- | --- | --- | --- | --- | --- | --- | --- | --- | --- | --- | --- | --- | --- |
|  | PrimeScript 1 Step Enzyme Mix | 5 U/μL TaKaRa Ex Taq HS | PrimeScript RT Enzyme Mix Ⅱ | aMPV-A | | | aMPV-B | | | aMPV-C | | | aMPV-D | | | RNase Free dH_2_O | RNA template | Total |
|  |  |  |  | F | R | P | F | R | P | F | R | P | F | R | P |  |  |  |
| 1 | 25.0 | 1.0 | 1.0 | 0.6 | 0.6 | 0.6 | 0.6 | 0.6 | 0.6 | 0.6 | 0.6 | 0.6 | 0.6 | 0.6 | 0.6 | 11.8 | 4.0 | 50 |
| 2 | 25.0 | 1.0 | 1.0 | 0.6 | 0.6 | 0.6 | 0.6 | 0.6 | 0.6 | 0.6 | 0.6 | 0.6 | 1.0 | 1.0 | 1.0 | 10.6 | 4.0 | 50 |
| 3 | 25.0 | 1.0 | 1.0 | 0.6 | 0.6 | 0.6 | 0.6 | 0.6 | 0.6 | 0.6 | 0.6 | 0.6 | 1.4 | 1.4 | 1.4 | 9.4 | 4.0 | 50 |
| 4 | 25.0 | 1.0 | 1.0 | 0.6 | 0.6 | 0.6 | 0.6 | 0.6 | 0.6 | 1.0 | 1.0 | 1.0 | 0.6 | 0.6 | 0.6 | 10.6 | 4.0 | 50 |
| 5 | 25.0 | 1.0 | 1.0 | 0.6 | 0.6 | 0.6 | 0.6 | 0.6 | 0.6 | 1.0 | 1.0 | 1.0 | 1.0 | 1.0 | 1.0 | 9.4 | 4.0 | 50 |
| 6 | 25.0 | 1.0 | 1.0 | 0.6 | 0.6 | 0.6 | 0.6 | 0.6 | 0.6 | 1.0 | 1.0 | 1.0 | 1.4 | 1.4 | 1.4 | 8.2 | 4.0 | 50 |
| 7 | 25.0 | 1.0 | 1.0 | 0.6 | 0.6 | 0.6 | 0.6 | 0.6 | 0.6 | 1.4 | 1.4 | 1.4 | 0.6 | 0.6 | 0.6 | 9.4 | 4.0 | 50 |
| 8 | 25.0 | 1.0 | 1.0 | 0.6 | 0.6 | 0.6 | 0.6 | 0.6 | 0.6 | 1.4 | 1.4 | 1.4 | 1.0 | 1.0 | 1.0 | 8.2 | 4.0 | 50 |
| 9 | 25.0 | 1.0 | 1.0 | 0.6 | 0.6 | 0.6 | 0.6 | 0.6 | 0.6 | 1.4 | 1.4 | 1.4 | 1.4 | 1.4 | 1.4 | 7.0 | 4.0 | 50 |
| 10 | 25.0 | 1.0 | 1.0 | 0.6 | 0.6 | 0.6 | 1.0 | 1.0 | 1.0 | 0.6 | 0.6 | 0.6 | 0.6 | 0.6 | 0.6 | 10.6 | 4.0 | 50 |
| 11 | 25.0 | 1.0 | 1.0 | 0.6 | 0.6 | 0.6 | 1.0 | 1.0 | 1.0 | 0.6 | 0.6 | 0.6 | 1.0 | 1.0 | 1.0 | 9.4 | 4.0 | 50 |
| 12 | 25.0 | 1.0 | 1.0 | 0.6 | 0.6 | 0.6 | 1.0 | 1.0 | 1.0 | 0.6 | 0.6 | 0.6 | 1.4 | 1.4 | 1.4 | 8.2 | 4.0 | 50 |
| 13 | 25.0 | 1.0 | 1.0 | 0.6 | 0.6 | 0.6 | 1.0 | 1.0 | 1.0 | 1.0 | 1.0 | 1.0 | 0.6 | 0.6 | 0.6 | 9.4 | 4.0 | 50 |
| 14 | 25.0 | 1.0 | 1.0 | 0.6 | 0.6 | 0.6 | 1.0 | 1.0 | 1.0 | 1.0 | 1.0 | 1.0 | 1.0 | 1.0 | 1.0 | 8.2 | 4.0 | 50 |
| 15 | 25.0 | 1.0 | 1.0 | 0.6 | 0.6 | 0.6 | 1.0 | 1.0 | 1.0 | 1.0 | 1.0 | 1.0 | 1.4 | 1.4 | 1.4 | 7.0 | 4.0 | 50 |
| 16 | 25.0 | 1.0 | 1.0 | 0.6 | 0.6 | 0.6 | 1.0 | 1.0 | 1.0 | 1.4 | 1.4 | 1.4 | 0.6 | 0.6 | 0.6 | 8.2 | 4.0 | 50 |
| 17 | 25.0 | 1.0 | 1.0 | 0.6 | 0.6 | 0.6 | 1.0 | 1.0 | 1.0 | 1.4 | 1.4 | 1.4 | 1.0 | 1.0 | 1.0 | 7.0 | 4.0 | 50 |
| 18 | 25.0 | 1.0 | 1.0 | 0.6 | 0.6 | 0.6 | 1.0 | 1.0 | 1.0 | 1.4 | 1.4 | 1.4 | 1.4 | 1.4 | 1.4 | 5.8 | 4.0 | 50 |
| 19 | 25.0 | 1.0 | 1.0 | 0.6 | 0.6 | 0.6 | 1.4 | 1.4 | 1.4 | 0.6 | 0.6 | 0.6 | 0.6 | 0.6 | 0.6 | 9.4 | 4.0 | 50 |
| 20 | 25.0 | 1.0 | 1.0 | 0.6 | 0.6 | 0.6 | 1.4 | 1.4 | 1.4 | 0.6 | 0.6 | 0.6 | 1.0 | 1.0 | 1.0 | 8.2 | 4.0 | 50 |
| 21 | 25.0 | 1.0 | 1.0 | 0.6 | 0.6 | 0.6 | 1.4 | 1.4 | 1.4 | 0.6 | 0.6 | 0.6 | 1.4 | 1.4 | 1.4 | 7.0 | 4.0 | 50 |
| 22 | 25.0 | 1.0 | 1.0 | 0.6 | 0.6 | 0.6 | 1.4 | 1.4 | 1.4 | 1.0 | 1.0 | 1.0 | 0.6 | 0.6 | 0.6 | 8.2 | 4.0 | 50 |
| 23 | 25.0 | 1.0 | 1.0 | 0.6 | 0.6 | 0.6 | 1.4 | 1.4 | 1.4 | 1.0 | 1.0 | 1.0 | 1.0 | 1.0 | 1.0 | 7.0 | 4.0 | 50 |
| 24 | 25.0 | 1.0 | 1.0 | 0.6 | 0.6 | 0.6 | 1.4 | 1.4 | 1.4 | 1.0 | 1.0 | 1.0 | 1.4 | 1.4 | 1.4 | 5.8 | 4.0 | 50 |
| 25 | 25.0 | 1.0 | 1.0 | 0.6 | 0.6 | 0.6 | 1.4 | 1.4 | 1.4 | 1.4 | 1.4 | 1.4 | 0.6 | 0.6 | 0.6 | 7.0 | 4.0 | 50 |
| 26 | 25.0 | 1.0 | 1.0 | 0.6 | 0.6 | 0.6 | 1.4 | 1.4 | 1.4 | 1.4 | 1.4 | 1.4 | 1.0 | 1.0 | 1.0 | 5.8 | 4.0 | 50 |
| 27 | 25.0 | 1.0 | 1.0 | 0.6 | 0.6 | 0.6 | 1.4 | 1.4 | 1.4 | 1.4 | 1.4 | 1.4 | 1.4 | 1.4 | 1.4 | 4.6 | 4.0 | 50 |
| 28 | 25.0 | 1.0 | 1.0 | 1.0 | 1.0 | 1.0 | 0.6 | 0.6 | 0.6 | 0.6 | 0.6 | 0.6 | 0.6 | 0.6 | 0.6 | 10.6 | 4.0 | 50 |
| 29 | 25.0 | 1.0 | 1.0 | 1.0 | 1.0 | 1.0 | 0.6 | 0.6 | 0.6 | 0.6 | 0.6 | 0.6 | 1.0 | 1.0 | 1.0 | 9.4 | 4.0 | 50 |
| 30 | 25.0 | 1.0 | 1.0 | 1.0 | 1.0 | 1.0 | 0.6 | 0.6 | 0.6 | 0.6 | 0.6 | 0.6 | 1.4 | 1.4 | 1.4 | 8.2 | 4.0 | 50 |
| 31 | 25.0 | 1.0 | 1.0 | 1.0 | 1.0 | 1.0 | 0.6 | 0.6 | 0.6 | 1.0 | 1.0 | 1.0 | 0.6 | 0.6 | 0.6 | 9.4 | 4.0 | 50 |
| 32 | 25.0 | 1.0 | 1.0 | 1.0 | 1.0 | 1.0 | 0.6 | 0.6 | 0.6 | 1.0 | 1.0 | 1.0 | 1.0 | 1.0 | 1.0 | 8.2 | 4.0 | 50 |
| 33 | 25.0 | 1.0 | 1.0 | 1.0 | 1.0 | 1.0 | 0.6 | 0.6 | 0.6 | 1.0 | 1.0 | 1.0 | 1.4 | 1.4 | 1.4 | 7.0 | 4.0 | 50 |
| 34 | 25.0 | 1.0 | 1.0 | 1.0 | 1.0 | 1.0 | 0.6 | 0.6 | 0.6 | 1.4 | 1.4 | 1.4 | 0.6 | 0.6 | 0.6 | 8.2 | 4.0 | 50 |
| 35 | 25.0 | 1.0 | 1.0 | 1.0 | 1.0 | 1.0 | 0.6 | 0.6 | 0.6 | 1.4 | 1.4 | 1.4 | 1.0 | 1.0 | 1.0 | 7.0 | 4.0 | 50 |
| 36 | 25.0 | 1.0 | 1.0 | 1.0 | 1.0 | 1.0 | 0.6 | 0.6 | 0.6 | 1.4 | 1.4 | 1.4 | 1.4 | 1.4 | 1.4 | 5.8 | 4.0 | 50 |
| 37 | 25.0 | 1.0 | 1.0 | 1.0 | 1.0 | 1.0 | 1.0 | 1.0 | 1.0 | 0.6 | 0.6 | 0.6 | 0.6 | 0.6 | 0.6 | 9.4 | 4.0 | 50 |
| 38 | 25.0 | 1.0 | 1.0 | 1.0 | 1.0 | 1.0 | 1.0 | 1.0 | 1.0 | 0.6 | 0.6 | 0.6 | 1.0 | 1.0 | 1.0 | 8.2 | 4.0 | 50 |
| 39 | 25.0 | 1.0 | 1.0 | 1.0 | 1.0 | 1.0 | 1.0 | 1.0 | 1.0 | 0.6 | 0.6 | 0.6 | 1.4 | 1.4 | 1.4 | 7.0 | 4.0 | 50 |
| 40 | 25.0 | 1.0 | 1.0 | 1.0 | 1.0 | 1.0 | 1.0 | 1.0 | 1.0 | 1.0 | 1.0 | 1.0 | 0.6 | 0.6 | 0.6 | 8.2 | 4.0 | 50 |
| 41 | 25.0 | 1.0 | 1.0 | 1.0 | 1.0 | 1.0 | 1.0 | 1.0 | 1.0 | 1.0 | 1.0 | 1.0 | 1.0 | 1.0 | 1.0 | 7.0 | 4.0 | 50 |
| 42 | 25.0 | 1.0 | 1.0 | 1.0 | 1.0 | 1.0 | 1.0 | 1.0 | 1.0 | 1.0 | 1.0 | 1.0 | 1.4 | 1.4 | 1.4 | 5.8 | 4.0 | 50 |
| 43 | 25.0 | 1.0 | 1.0 | 1.0 | 1.0 | 1.0 | 1.0 | 1.0 | 1.0 | 1.4 | 1.4 | 1.4 | 0.6 | 0.6 | 0.6 | 7.0 | 4.0 | 50 |
| 44 | 25.0 | 1.0 | 1.0 | 1.0 | 1.0 | 1.0 | 1.0 | 1.0 | 1.0 | 1.4 | 1.4 | 1.4 | 1.0 | 1.0 | 1.0 | 5.8 | 4.0 | 50 |
| 45 | 25.0 | 1.0 | 1.0 | 1.0 | 1.0 | 1.0 | 1.0 | 1.0 | 1.0 | 1.4 | 1.4 | 1.4 | 1.4 | 1.4 | 1.4 | 4.6 | 4.0 | 50 |
| 46 | 25.0 | 1.0 | 1.0 | 1.0 | 1.0 | 1.0 | 1.4 | 1.4 | 1.4 | 0.6 | 0.6 | 0.6 | 0.6 | 0.6 | 0.6 | 8.2 | 4.0 | 50 |
| 47 | 25.0 | 1.0 | 1.0 | 1.0 | 1.0 | 1.0 | 1.4 | 1.4 | 1.4 | 0.6 | 0.6 | 0.6 | 1.0 | 1.0 | 1.0 | 7.0 | 4.0 | 50 |
| 48 | 25.0 | 1.0 | 1.0 | 1.0 | 1.0 | 1.0 | 1.4 | 1.4 | 1.4 | 0.6 | 0.6 | 0.6 | 1.4 | 1.4 | 1.4 | 5.8 | 4.0 | 50 |
| 49 | 25.0 | 1.0 | 1.0 | 1.0 | 1.0 | 1.0 | 1.4 | 1.4 | 1.4 | 1.0 | 1.0 | 1.0 | 0.6 | 0.6 | 0.6 | 7.0 | 4.0 | 50 |
| 50 | 25.0 | 1.0 | 1.0 | 1.0 | 1.0 | 1.0 | 1.4 | 1.4 | 1.4 | 1.0 | 1.0 | 1.0 | 1.0 | 1.0 | 1.0 | 5.8 | 4.0 | 50 |
| 51 | 25.0 | 1.0 | 1.0 | 1.0 | 1.0 | 1.0 | 1.4 | 1.4 | 1.4 | 1.0 | 1.0 | 1.0 | 1.4 | 1.4 | 1.4 | 4.6 | 4.0 | 50 |
| 52 | 25.0 | 1.0 | 1.0 | 1.0 | 1.0 | 1.0 | 1.4 | 1.4 | 1.4 | 1.4 | 1.4 | 1.4 | 0.6 | 0.6 | 0.6 | 5.8 | 4.0 | 50 |
| 53 | 25.0 | 1.0 | 1.0 | 1.0 | 1.0 | 1.0 | 1.4 | 1.4 | 1.4 | 1.4 | 1.4 | 1.4 | 1.0 | 1.0 | 1.0 | 4.6 | 4.0 | 50 |
| 54 | 25.0 | 1.0 | 1.0 | 1.0 | 1.0 | 1.0 | 1.4 | 1.4 | 1.4 | 1.4 | 1.4 | 1.4 | 1.4 | 1.4 | 1.4 | 3.4 | 4.0 | 50 |
| 55 | 25.0 | 1.0 | 1.0 | 1.4 | 1.4 | 1.4 | 0.6 | 0.6 | 0.6 | 0.6 | 0.6 | 0.6 | 0.6 | 0.6 | 0.6 | 9.4 | 4.0 | 50 |
| 56 | 25.0 | 1.0 | 1.0 | 1.4 | 1.4 | 1.4 | 0.6 | 0.6 | 0.6 | 0.6 | 0.6 | 0.6 | 1.0 | 1.0 | 1.0 | 8.2 | 4.0 | 50 |
| 57 | 25.0 | 1.0 | 1.0 | 1.4 | 1.4 | 1.4 | 0.6 | 0.6 | 0.6 | 0.6 | 0.6 | 0.6 | 1.4 | 1.4 | 1.4 | 7.0 | 4.0 | 50 |
| 58 | 25.0 | 1.0 | 1.0 | 1.4 | 1.4 | 1.4 | 0.6 | 0.6 | 0.6 | 1.0 | 1.0 | 1.0 | 0.6 | 0.6 | 0.6 | 8.2 | 4.0 | 50 |
| 59 | 25.0 | 1.0 | 1.0 | 1.4 | 1.4 | 1.4 | 0.6 | 0.6 | 0.6 | 1.0 | 1.0 | 1.0 | 1.0 | 1.0 | 1.0 | 7.0 | 4.0 | 50 |
| 60 | 25.0 | 1.0 | 1.0 | 1.4 | 1.4 | 1.4 | 0.6 | 0.6 | 0.6 | 1.0 | 1.0 | 1.0 | 1.4 | 1.4 | 1.4 | 5.8 | 4.0 | 50 |
| 61 | 25.0 | 1.0 | 1.0 | 1.4 | 1.4 | 1.4 | 0.6 | 0.6 | 0.6 | 1.4 | 1.4 | 1.4 | 0.6 | 0.6 | 0.6 | 7.0 | 4.0 | 50 |
| 62 | 25.0 | 1.0 | 1.0 | 1.4 | 1.4 | 1.4 | 0.6 | 0.6 | 0.6 | 1.4 | 1.4 | 1.4 | 1.0 | 1.0 | 1.0 | 5.8 | 4.0 | 50 |
| 63 | 25.0 | 1.0 | 1.0 | 1.4 | 1.4 | 1.4 | 0.6 | 0.6 | 0.6 | 1.4 | 1.4 | 1.4 | 1.4 | 1.4 | 1.4 | 4.6 | 4.0 | 50 |
| 64 | 25.0 | 1.0 | 1.0 | 1.4 | 1.4 | 1.4 | 1.0 | 1.0 | 1.0 | 0.6 | 0.6 | 0.6 | 0.6 | 0.6 | 0.6 | 8.2 | 4.0 | 50 |
| 65 | 25.0 | 1.0 | 1.0 | 1.4 | 1.4 | 1.4 | 1.0 | 1.0 | 1.0 | 0.6 | 0.6 | 0.6 | 1.0 | 1.0 | 1.0 | 7.0 | 4.0 | 50 |
| 66 | 25.0 | 1.0 | 1.0 | 1.4 | 1.4 | 1.4 | 1.0 | 1.0 | 1.0 | 0.6 | 0.6 | 0.6 | 1.4 | 1.4 | 1.4 | 5.8 | 4.0 | 50 |
| 67 | 25.0 | 1.0 | 1.0 | 1.4 | 1.4 | 1.4 | 1.0 | 1.0 | 1.0 | 1.0 | 1.0 | 1.0 | 0.6 | 0.6 | 0.6 | 7.0 | 4.0 | 50 |
| 68 | 25.0 | 1.0 | 1.0 | 1.4 | 1.4 | 1.4 | 1.0 | 1.0 | 1.0 | 1.0 | 1.0 | 1.0 | 1.0 | 1.0 | 1.0 | 5.8 | 4.0 | 50 |
| 69 | 25.0 | 1.0 | 1.0 | 1.4 | 1.4 | 1.4 | 1.0 | 1.0 | 1.0 | 1.0 | 1.0 | 1.0 | 1.4 | 1.4 | 1.4 | 4.6 | 4.0 | 50 |
| 70 | 25.0 | 1.0 | 1.0 | 1.4 | 1.4 | 1.4 | 1.0 | 1.0 | 1.0 | 1.4 | 1.4 | 1.4 | 0.6 | 0.6 | 0.6 | 5.8 | 4.0 | 50 |
| 71 | 25.0 | 1.0 | 1.0 | 1.4 | 1.4 | 1.4 | 1.0 | 1.0 | 1.0 | 1.4 | 1.4 | 1.4 | 1.0 | 1.0 | 1.0 | 4.6 | 4.0 | 50 |
| 72 | 25.0 | 1.0 | 1.0 | 1.4 | 1.4 | 1.4 | 1.0 | 1.0 | 1.0 | 1.4 | 1.4 | 1.4 | 1.4 | 1.4 | 1.4 | 3.4 | 4.0 | 50 |
| 73 | 25.0 | 1.0 | 1.0 | 1.4 | 1.4 | 1.4 | 1.4 | 1.4 | 1.4 | 0.6 | 0.6 | 0.6 | 0.6 | 0.6 | 0.6 | 7.0 | 4.0 | 50 |
| 74 | 25.0 | 1.0 | 1.0 | 1.4 | 1.4 | 1.4 | 1.4 | 1.4 | 1.4 | 0.6 | 0.6 | 0.6 | 1.0 | 1.0 | 1.0 | 5.8 | 4.0 | 50 |
| 75 | 25.0 | 1.0 | 1.0 | 1.4 | 1.4 | 1.4 | 1.4 | 1.4 | 1.4 | 0.6 | 0.6 | 0.6 | 1.4 | 1.4 | 1.4 | 4.6 | 4.0 | 50 |
| 76 | 25.0 | 1.0 | 1.0 | 1.4 | 1.4 | 1.4 | 1.4 | 1.4 | 1.4 | 1.0 | 1.0 | 1.0 | 0.6 | 0.6 | 0.6 | 5.8 | 4.0 | 50 |
| 77 | 25.0 | 1.0 | 1.0 | 1.4 | 1.4 | 1.4 | 1.4 | 1.4 | 1.4 | 1.0 | 1.0 | 1.0 | 1.0 | 1.0 | 1.0 | 4.6 | 4.0 | 50 |
| 78 | 25.0 | 1.0 | 1.0 | 1.4 | 1.4 | 1.4 | 1.4 | 1.4 | 1.4 | 1.0 | 1.0 | 1.0 | 1.4 | 1.4 | 1.4 | 3.4 | 4.0 | 50 |
| 79 | 25.0 | 1.0 | 1.0 | 1.4 | 1.4 | 1.4 | 1.4 | 1.4 | 1.4 | 1.4 | 1.4 | 1.4 | 0.6 | 0.6 | 0.6 | 4.6 | 4.0 | 50 |
| 80 | 25.0 | 1.0 | 1.0 | 1.4 | 1.4 | 1.4 | 1.4 | 1.4 | 1.4 | 1.4 | 1.4 | 1.4 | 1.0 | 1.0 | 1.0 | 3.4 | 4.0 | 50 |
| 81 | 25.0 | 1.0 | 1.0 | 1.4 | 1.4 | 1.4 | 1.4 | 1.4 | 1.4 | 1.4 | 1.4 | 1.4 | 1.4 | 1.4 | 1.4 | 2.2 | 4.0 | 50 |
