# Supplementary material for: Establishment and application of a quadruple real-time RT-PCR for detecting avian metapneumovirus
Source: PLoS One. 2022 Jun 28;17(6):e0270708. doi: 10.1371/journal.pone.0270708 (PMC9239461; doi:10.1371/journal.pone.0270708)
Supplement: S3 Table — The quadruple real-time RT-PCR reaction system was performed according to the BioScript (Dalian) Co., Ltd. PrimeScriptTM One Step RT-PCR Kit instructions. The standard recombinant plasmids for the four subgroups (104 cRNA copies per reaction) were used as templates. The mean values of CT were calculated to analyze and screen the optimal volume of primers and probes. (DOCX) [file pone.0270708.s003.docx]

**S3 Table The test results of different primers and probes volume in the reaction mixture**

| number | Cycle threshold values | | | | | | | | | | | | | | | |
| --- | --- | --- | --- | --- | --- | --- | --- | --- | --- | --- | --- | --- | --- | --- | --- | --- |
|  | aMPV-A | | | | aMPV-B | | | | aMPV-C | | | | aMPV-D | | | |
|  | ROX | FAM | VIC | CY5 | ROX | FAM | VIC | CY5 | ROX | FAM | VIC | CY5 | ROX | FAM | VIC | CY5 |
| 1 | 28.79 | - | - | - | - | 25.88 | - | - | - | - | 26.66 | - | - | - | - | 29.31 |
| 2 | 28.97 | - | - | - | - | 25.25 | - | - | - | - | 26.97 | - | - | - | - | 29.24 |
| 3 | 29.21 | - | - | - | - | 25.66 | - | - | - | - | 26.95 | - | - | - | - | 28.74 |
| 4 | 28.68 | - | - | - | - | 25.25 | 38.93 | - | - | - | 26.17 | - | - | - | - | 29.55 |
| 5 | 28.85 | - | - | - | - | 25.76 | - | - | - | - | 26.56 | - | - | - | - | 29.16 |
| 6 | 28.95 | - | - | - | - | 25.50 | - | - | - | - | 26.11 | - | - | - | - | 29.04 |
| 7 | 29.38 | - | - | - | - | 25.75 | 39.81 | - | - | - | 25.89 | - | - | - | - | 29.94 |
| 8 | 28.76 | - | - | - | - | 25.34 | - | - | - | - | 25.10 | - | - | - | - | 29.23 |
| 9 | - | - | - | - | - | 25.37 | - | - | - | - | 25.39 | - | - | - | - | 29.34 |
| 10 | 26.90 | - | - | - | - | 24.96 | - | - | - | - | 26.25 | - | - | - | - | 29.26 |
| 11 | 26.88 | - | - | - | - | 25.68 | - | - | - | - | 26.86 | - | - | - | - | 29.59 |
| 12 | 27.54 | - | - | - | - | 25.05 | - | - | - | - | 27.37 | - | - | - | - | 29.73 |
| 13 | - | - | - | - | - | 25.65 | - | - | - | - | 25.83 | - | - | - | - | 29.01 |
| 14 | 27.87 | - | - | - | - | 25.22 | - | - | - | - | 26.13 | - | - | - | - | 29.85 |
| 15 | 27.51 | - | - | - | - | 25.30 | - | - | - | - | 25.00 | - | - | - | - | 29.23 |
| 16 | 27.53 | - | - | - | - | 25.36 | 39.01 | - | - | - | 25.39 | - | - | - | - | 29.66 |
| 17 | 28.51 | - | - | - | - | 25.78 | - | - | - | - | 25.71 | - | - | - | - | 28.92 |
| 18 | 27.35 | - | - | - | - | 25.56 | - | - | - | - | 25.58 | - | - | - | - | 29.87 |
| 19 | 27.98 |  |  |  |  | 25.57 | - | - | - | - | 26.51 | - | - | - | - | 28.75 |
| 20 | - |  |  |  |  | - | - | - | - | - | 27.19 | - | - | - | - | 29.22 |
| 21 | 27.19 |  |  |  | - | 25.33 | - | - | - | - | 28.02 | - | - | - | - | 28.71 |
| 22 | 27.38 | - | - | - | - | 25.17 | 39.05 |  |  | - | 27.77 | - | - | - | - | 28.89 |
| 23 | 27.12 | - | - | - | - | 25.26 | - | - | - | - | 27.62 | - | - | - | - | 28.77 |
| 24 | 27.12 | - | - | - | - | 25.10 | - | - | - | - | 27.80 | - | - | - | - | 29.21 |
| 25 | - | - | - | - | - | 25.32 | - | - | - | - | 26.72 | - | - | - | - | 28.92 |
| 26 | 27.52 | - | - | - | - | - | - | - | - | - | 27.27 | - | - | - | - | 28.78 |
| 27 | 27.35 | - | - | - | - | 25.37 | - | - | - | - | 27.74 | - | - | - | - | 28.36 |
| 28 | 29.62 | - | - | - | - | 25.15 | - | - | - | - | 27.40 | - | - | - | - | 29.15 |
| 29 | 29.89 | - | - | - | - | 25.23 | - | - | - | - | 28.38 | - | - | - | - | 28.82 |
| 30 | 29.75 | - | - | - | - | 24.98 | - | - | - | - | 28.17 | - | - | - | - | 29.20 |
| 31 | 29.59 | - | - | - | - | 25.19 | - | - | - | - | 27.68 | - | - | - | - | 28.91 |
| 32 | 31.89 | - | - | - | - | 24.59 | - | - | - | - | 27.88 | - | - | - | - | 28.88 |
| 33 | 29.22 | - | - | - | - | 25.21 | - | - | - | - | 27.75 | - | - | - | - | 28.73 |
| 34 | 29.91 | - | - | - | - | 25.05 | - | - | - | - | 27.15 | - | - | - | - | 27.66 |
| 35 | 29.31 | - | - | - | - | 25.44 | - | - | - | - | 28.00 | - | - | - | - | 29.10 |
| 36 | 29.00 | - | - | - | - | 25.13 | - | - | - | - | 27.32 | - | - | - | - | 29.23 |
| 37 | 27.15 | - | - | - | - | 25.55 | - | - | - | - | 27.71 | - | - | - | - | 29.19 |
| 38 | 27.88 | - | - | - | - | 25.04 | - | - | - | - | 26.77 | - | - | - | - | 29.56 |
| 39 | 28.09 | - | - | - | - | 25.40 | - | - | - | - | 27.44 | - | - | - | - | 29.19 |
| 40 | 27.94 | - | - | - | - | 25.32 | - | - | - | - | #VALUE! | - | - | - | - | 29.01 |
| 41 | 27.89 | - | - | - | - | 25.11 | - | - | - | - | 27.51 | - | - | - | - | 29.83 |
| 42 | 27.74 | - | - | - | - | 24.52 | 37.74 | - | - | - | 27.89 | - | - | - | - | 29.55 |
| 43 | 28.80 | - | - | - | - | 25.26 | - | - | - | - | 27.45 | - | - | - | - | 29.26 |
| 44 | 27.80 | - | - | - | - | 24.95 | - | - | - | - | 27.82 | - | - | - | - | 29.80 |
| 45 | 28.19 | - | - | - | - | 24.98 | - | - | - | - | 27.67 | 38.74 | - | - | - | 28.80 |
| 46 | 27.95 | - | - | - | - | 24.53 | 37.96 | - | - | - | 27.93 | - | - | - | - | 29.25 |
| 47 | 27.39 | - | - | - | - | 24.92 | - | - | - | - | 28.40 | - | - | - | - | 29.10 |
| 48 | 27.33 | - | - | - | - | 24.58 | - | - | - | - | 28.40 | - | - | - | - | 28.71 |
| 49 | 27.36 | - | - | - | - | 24.89 | - | - | - | - | 28.62 | - | - | - | - | 28.63 |
| 50 | 27.59 | - | - | - | - | 24.96 | 37.66 | - | - | - | 27.41 | - | - | 39.38 | 39.32 | 28.75 |
| 51 | 27.44 | - | - | - | - | 25.34 | - | - | - | - | 27.28 | - | - | - | - | 28.32 |
| 52 | 27.35 | - | - | - | - | 24.96 | - | - | - | - | 27.52 | - | - | - | - | 29.00 |
| 53 | 27.79 | - | - | - | - | 25.17 | - | - | - | - | 27.30 | - | - | - | - | 27.82 |
| 54 | 27.86 | - | - | - | - | 24.79 | - | - | - | - | 27.46 | - | - | - | - | 28.03 |
| 55 | 28.12 | - | - | - | - | 24.87 | - | - | - | - | 27.60 | - | - | - | - | 28.43 |
| 56 | 29.24 | - | - | - | - | 25.09 | - | - | - | - | 27.92 | - | - | - | - | 29.04 |
| 57 | 28.32 | - | - | - | - | 25.06 | - | - | - | - | 27.80 | - | - | - | - | 28.97 |
| 58 | 29.91 | - | - | - | - | 24.53 | - | - | - | - | 27.57 | - | - | - | - | 28.91 |
| 59 | 28.18 | - | - | - | - | 25.01 | - | - | - | - | 27.46 | - | - | - | - | 28.46 |
| 60 | 29.40 | - | - | - | - | 24.70 | - | - | - | - | 27.44 | - | - | - | - | 29.10 |
| 61 | 28.93 | - | - | - | - | 25.22 | - | - | - | - | 27.22 | - | - | - | - | 28.89 |
| 62 | 29.07 | - | - | - | - | 24.93 | - | - | - | - | 27.64 | - | - | - | - | 28.88 |
| 63 | 28.89 | - | - | - | - | 24.74 | - | - | - | - | 27.34 | - | - | - | - | 29.08 |
| 64 | 27.24 | - | - | - | - | 24.89 | - | - | - | - | 27.41 | - | - | - | - | 29.27 |
| 65 | 27.36 | - | - | - | - | 24.96 | - | - | - | - | 27.33 | - | - | - | - | 29.37 |
| 66 | 27.45 | - | - | - | - | 24.92 | - | - | - | - | 27.45 | - | - | - | - | 29.27 |
| 67 | 27.52 | - | - | - | - | 24.90 | - | - | - | - | 26.38 | - | - | - | - | 27.56 |
| 68 | 27.20 | - | - | - | - | 24.57 | - | - | - | - | 27.03 | - | - | - | - | 28.06 |
| 69 | 27.36 | - | - | - | - | - | - | - | - | - | 26.57 | - | - | - | - | 29.67 |
| 70 | 26.97 | - | - | - | - | 24.76 | - | - | - | - | 27.79 | - | - | - | - | 29.47 |
| 71 | 27.65 | - | - | - | - | 25.05 | - | - | - | - | 27.36 | - | - | - | - | 29.27 |
| 72 | 27.50 | - | - | - | - | 24.52 | - | - | - | - | 27.12 | - | - | - | - | 29.41 |
| 73 | 26.77 | - | - | - | - | 24.87 | - | - | - | - | 27.57 | - | - | - | - | 29.15 |
| 74 | 26.79 | - | - | - | - | 24.84 | - | - | - | - | 27.51 | - | - | - | - | 29.08 |
| 75 | 27.00 | - | - | - | - | 25.08 | - | - | - | - | 27.33 | - | - | - | - | 28.57 |
| 76 | 26.93 | - | - | - | - | 24.65 | - | - | - | - | 26.81 | - | - | - | - | - |
| 77 | 27.98 | - | - | - | - | 26.02 | - | - | - | - | 27.43 | - | - | - | - | 28.31 |
| 78 | 27.44 | - | - | - | - | 24.86 | - | - | - | - | 26.90 | - | - | - | - | 28.69 |
| 79 | 27.27 | - | - | - | - | 25.13 | - | - | - | - | 27.25 | - | - | - | - | 28.95 |
| 80 | 27.18 | - | - | - | - | - | - | - | - | - | 26.85 | - | - | - | - | 28.76 |
| 81 | 27.59 | - | - | - | - | 25.26 | - | - | - | - | 26.87 | - | - | - | - | 28.79 |

The quadruple real-time RT-PCR reaction system was performed according to the BioScript (Dalian) Co., Ltd. PrimeScriptTM One Step RT-PCR Kit instructions. The standard recombinant plasmids for the four subgroups (10^4^ cRNA copies per reaction) were used as templates. The mean values of CT were calculated to analyze and screen the optimal volume of primers and probes.
