# Supplementary material for: Establishment and application of a quadruple real-time RT-PCR for detecting avian metapneumovirus
Source: PLoS One. 2022 Jun 28;17(6):e0270708. doi: 10.1371/journal.pone.0270708 (PMC9239461; doi:10.1371/journal.pone.0270708)
Supplement: S4 Table — The results showed that the mean values of CT were decreasing with the increasing of primer and probes volumes. However, when the volume of primers and probes increased from 1.0μL to 1.4μL, the CT values dose not decreased significantly. Therefore, considering the cost and results of the experiment, the volume of primers and probes for the four subgroups were set as 1.0μL in the 50μL reaction mixture. The reaction mixture contained the following: 25.0 μL of 2× One Step RT-PCR Buffer Ⅲ, 1.0 μL of 5 U/μL TaKaRa Ex Taq HS, 1.0 μL of PrimeScript RT Enzyme Mix Ⅱ, 1.0 μL of the primers and probes for each subgroup of aMPV (10 pmol/μL), 4.0 μL of RNA template and RNase Free dH2O to a final volume of 50.0 μL. (DOCX) [file pone.0270708.s004.docx]

**S4 Table The mean values of cycle threshold**

| Volumes (µL) | The mean values of cycle threshold | | | |
| --- | --- | --- | --- | --- |
|  | aMPV-A | aMPV-B | aMPV-C | aMPV-D |
| 0.6 | 29.22 | 25.42 | 27.46 | 29.40 |
| 1.0 | 27.61 | 25.06 | 27.10 | 28.94 |
| 1.4 | 27.39 | 24.94 | 26.96 | 28.90 |

The results showed that the mean values of CT were decreasing with the increasing of primer and probes volumes. However, when the volume of primers and probes increased from 1.0µL to 1.4µL, the CT values dose not decreased significantly. Therefore, considering the cost and results of the experiment, the volume of primers and probes for the four subgroups were set as 1.0µL in the 50µL reaction mixture. The reaction mixture contained the following: 25.0 µL of 2× One Step RT-PCR Buffer Ⅲ, 1.0 µL of 5 U/μL TaKaRa Ex Taq HS, 1.0 µL of PrimeScript RT Enzyme Mix Ⅱ, 1.0 µL of the primers and probes for each subgroup of aMPV (10 pmol/μL), 4.0 µL of RNA template and RNase Free dH_2_O to a final volume of 50.0 µL.
