# Supplementary material for: Establishment and application of a quadruple real-time RT-PCR for detecting avian metapneumovirus
Source: PLoS One. 2022 Jun 28;17(6):e0270708. doi: 10.1371/journal.pone.0270708 (PMC9239461; doi:10.1371/journal.pone.0270708)
Supplement: S5 Table — Five annealing/extension temperature (58°C, 59°C, 60°C, 61°C, 62°C) were tested to screen the optimal annealing/extension conditions. The results showed that CT values were smaller and the sensitivity was higher than other temperatures when the annealing/extension temperature was 59°C or 60°C. In the appropriate temperature range, the specificity of primers and probes will increase with the raising of annealing/extension temperature. Therefore, the annealing/extension temperature of the quadruple real-time RT-PCR was set as 60°C. (DOCX) [file pone.0270708.s005.docx]

**S5 Table The test results of optimum annealing/extension temperature for the quadruple real-time RT-PCR**

| cRNA copies per reaction | Cycle threshold | | | | | | | | | | | | | | | | | | | |  |
| --- | --- | --- | --- | --- | --- | --- | --- | --- | --- | --- | --- | --- | --- | --- | --- | --- | --- | --- | --- | --- | --- |
|  | 58°C | | | | 59°C | | | | 60°C | | | | 61°C | | | | 62°C | | | |  |
|  | ROX | FAM | VIC | CY5 | ROX | FAM | VIC | CY5 | ROX | FAM | VIC | CY5 | ROX | FAM | VIC | CY5 | ROX | FAM | VIC | CY5 | |
| 10^5^ | 25.55 | 21.91 | 23.51 | 25.47 | 25.41 | 22.01 | 23.61 | 25.59 | 25.48 | 22.11 | 23.52 | 25.60 | 25.50 | 22.36 | 24.16 | 25.88 | 25.73 | 22.39 | 24.31 | 26.05 | |
| 10^4^ | 28.13 | 24.92 | 27.75 | 29.57 | 28.66 | 25.16 | 27.77 | 29.00 | 28.24 | 25.53 | 27.72 | 29.20 | 28.20 | 26.29 | 27.94 | 29.55 | 28.38 | 26.30 | 28.78 | 29.89 | |
| 10^3^ | 33.61 | 28.93 | 31.46 | 33.52 | 31.74 | 29.09 | 31.71 | 32.45 | 31.58 | 29.64 | 31.53 | 32.11 | 31.82 | 29.71 | 31.57 | 32.32 | 31.63 | 30.51 | 32.47 | 32.59 | |
| 10^2^ | - | 33.01 | 35.83 | 37.35 | 36.01 | 33.55 | 35.04 | 36.81 | 36.58 | 33.97 | 35.01 | 36.31 | 38.41 | 34.84 | - | 37.12 | - | 36.91 | - | - | |

Five annealing/extension temperature (58°C, 59°C, 60°C, 61°C, 62°C) were tested to screen the optimal annealing/extension conditions. The results showed that CT values were smaller and the sensitivity was higher than other temperatures when the annealing/extension temperature was 59°C or 60°C. In the appropriate temperature range, the specificity of primers and probes will increase with the raising of annealing/extension temperature. Therefore, the annealing/extension temperature of the quadruple real-time RT-PCR was set as 60°C.
