# Supplementary material for: Establishment and application of a quadruple real-time RT-PCR for detecting avian metapneumovirus
Source: PLoS One. 2022 Jun 28;17(6):e0270708. doi: 10.1371/journal.pone.0270708 (PMC9239461; doi:10.1371/journal.pone.0270708)
Supplement: S6 Table — Three annealing/extension time (20s, 30s, 40s) were tested to screen the optimal annealing/extension conditions. The results showed that there was no significant difference in the CT values when the annealing/extension time was 20s, 30s and 40s respectively. The annealing/extension time was set as 30s to prevent the influence of stability of the experiment by too short annealing/extension time. The quadruple real-time RT-PCR cycler conditions were as follows: 30 min at 50°C, 4 min at 95°C and 40 cycles of 98°C for 30 s, 60°C for 30 s and 72°C for 30 s, followed by a final extension step for 7 min at 72°C. (DOCX) [file pone.0270708.s006.docx]

**S6 Table The test results of optimum annealing/extension time for the quadruple real-time RT-PCR**

| cRNA copies per reaction | Cycle threshold | | | | | | | | | | | |
| --- | --- | --- | --- | --- | --- | --- | --- | --- | --- | --- | --- | --- |
|  | 20s | | | | 30s | | | | 40s | | | |
|  | ROX | FAM | VIC | CY5 | ROX | FAM | VIC | CY5 | ROX | FAM | VIC | CY5 |
| 10^5^ | 25.68 | 22.31 | 24.64 | 25.12 | 25.94 | 22.04 | 24.32 | 24.46 | 26.48 | 22.17 | 24.71 | 25.19 |
|  | 25.88 | 22.29 | 24.71 | 25.04 | 25.39 | 22.52 | 24.59 | 24.49 | 25.89 | 22.39 | 24.43 | 25.33 |
|  | 25.46 | 22.36 | 25.11 | 24.99 | 25.67 | 21.85 | 24.84 | 24.22 | 26.06 | 22.26 | 24.55 | 24.27 |
| 10^4^ | 28.73 | 25.67 | 29.09 | 28.36 | 28.86 | 25.52 | 28.98 | 28.27 | 29.15 | 25.31 | 29.29 | 28.24 |
|  | 28.80 | 25.83 | 28.86 | 28.30 | 28.74 | 25.96 | 29.57 | 28.47 | 28.88 | 26.08 | 29.30 | 28.55 |
|  | 28.05 | 25.85 | 28.66 | 28.87 | 28.89 | 25.44 | 29.14 | 28.20 | 28.52 | 25.97 | 29.24 | 28.79 |
| 10^3^ | 32.48 | 31.36 | 32.00 | 31.09 | 32.48 | 29.50 | 32.89 | 31.89 | 32.38 | 29.65 | 33.74 | 33.15 |
|  | 32.48 | 30.91 | 33.36 | 29.36 | 32.01 | 29.72 | 33.52 | 31.77 | 32.00 | 29.98 | 33.44 | 32.97 |
|  | 32.07 | 30.40 | 33.03 | 30.16 | 32.24 | 30.42 | 32.65 | 32.06 | 32.78 | 28.46 | 34.66 | 33.24 |
| 10^2^ | 35.41 | 35.77 | - | 33.80 | 35.92 | 33.15 | 35.52 | 34.84 | 35.97 | 33.08 | 37.86 | 34.89 |
|  | 35.69 | 33.35 | 36.45 | 34.13 | 35.34 | 34.52 | - | 34.40 | 37.04 | 33.26 | 36.38 | 36.44 |
|  | 36.01 | 34.97 | - | 34.21 | 36.13 | 32.93 | 35.29 | 35.04 | 36.89 | 34.07 | - | 35.38 |

Three annealing/extension time (20s, 30s, 40s) were tested to screen the optimal annealing/extension conditions. The results showed that there was no significant difference in the CT values when the annealing/extension time was 20s, 30s and 40s respectively. The annealing/extension time was set as 30s to prevent the influence of stability of the experiment by too short annealing/extension time. The quadruple real-time RT‑PCR cycler conditions were as follows: 30 min at 50°C, 4 min at 95°C and 40 cycles of 98°C for 30 s, 60°C for 30 s and 72°C for 30 s, followed by a final extension step for 7 min at 72°C.
